# Comparison Between Micro- and Micro-Nano Surface Texturization in the Initial Osseointegration Process: An Experimental In Vitro and In Vivo Preclinical Study

**DOI:** 10.3390/bioengineering12020175

**Published:** 2025-02-12

**Authors:** Sergio Alexandre Gehrke, Eleani Maria da Costa, Jaime Aramburú Júnior, Tiago Luis Eilers Treichel, Massimo Del Fabbro, Antonio Scarano

**Affiliations:** 1 Department of Implantology, Bioface/Postgrados en Odontología/Universidad Catolica de Murcia, Montevideo 11100, Uruguay; jaimearamburujunior@gmail.com; 2 Department of Biotechnology, Universidad Católica de Murcia, 30107 Murcia, Spain; 3 Department of Materials Engineering, Pontificial Catholic University of Rio Grande do Sul, Porto Alegre 90619-900, Brazil; eleani@pucrs.br; 4 Department of Physiology and Pharmacology, Pro-Rectorate of Graduate Studies and Research (PRPGP) of the Universidade Federal de Santa Maria, Santa Maria 97105-900, Brazil; 5 Department of Surgery, Faculty of Medicine Veterinary, University of Rio Verde, Rio Verde 75901-970, Brazil; tiago@unirv.edu.br; 6 Department of Biomedical Surgical and Dental Sciences, University of Milan, 20122 Milan, Italy; massimo.delfabbro@unimi.it; 7 Fondazione IRCCS Ca’ Granda Ospedale Maggiore Policlinico, 20122 Milan, Italy; 8 Department of Innovative Technologies in Medicine & Dentistry, University of Chieti-Pescara, 66013 Chieti, Italy; ascarano@unich.it

**Keywords:** dental implants, in vitro and in vivo study, histomorphometry, implant stability, implant surface, micro- and micro-nano surface, rabbit animal study, bone–implant contact, tissue area fraction occupancy, implant removal torque

## Abstract

Background: The physicochemical changes of the surface aim to improve cell adhesion, proliferation, and differentiation, that is, better biological interaction with the cells and, consequently, with the peri-implant tissues. In the present study, implants with the same macrogeometry were compared in vitro and in vivo, but with two different surfaces: micro-rough and a new micro-nano-rough surface. Materials and Methods: A total of 90 implants were used, 10 of which were used for in vitro surface characterization (n = 5 per group) through scanning electron microscopy (SEM), atomic force microscopy (AFM), and surface roughness measurements. For in vivo tests, 80 implants (n = 40 per group) were used in 20 rabbits (n = 2 implants per tibia). Two experimental groups were created: a control group, where the implants had a surface treated by sandblasting with titanium oxide microparticles, and a test group, where the implants were sandblasted using the same process as the previous group plus acid conditioned. The implant stability quotient (ISQ) was measured by resonance frequency (initially and at both euthanasia times). Animals were euthanized 3 and 5 weeks after implantation (n = 10 animals per time). Ten samples from each group at each time point were evaluated by removal torque (RTv). Another ten samples from each group were evaluated histologically and histomorphometrically, measuring the percentage of bone-to-implant contact (%BIC) and the bone area fraction occupancy (%BAFO). Results: In vitro, it was possible to observe a more homogeneous surface for the test group compared to the control group. ISQ values showed statistical differences at both 3 and 5 weeks (test > control). For RTv, the values were: 44.5 ± 4.25 Ncm (control group) and 48.6 ± 3.17 Ncm (test group) for the time of 3 weeks; 64.3 ± 4.50 Ncm (control group) and 76.1 ± 4.18 Ncm (test group) at 5 weeks. The %BIC and %BAFO values measured in both groups and at both times did not show significant differences (*p* > 0.05). Conclusions: The higher removal torque and ISQ values presented in the samples from the test group compared to the control group indicate that there was an acceleration in the mineralization process of the newly formed bone matrix.

## 1. Introduction

Oral rehabilitation with implants has become a predictable treatment alternative and plays an important role in restoring masticatory function, aesthetics, and quality of life for patients who have lost teeth. Currently, there are many techniques for installing dental implants, plus increasingly advanced materials are used in this process, and knowledge of bone biology continues to evolve and leverage these technological developments [1,2]. Dental implants significantly influence the treatment of edentulism, as they have a considerably high survival rate through osseointegration, which is dependent on the interaction between the bone and the physicochemical properties of the implants [3].

Commercially pure titanium (cp Ti) has properties that allow its application as a biomaterial, such as spontaneous formation of a highly biocompatible Ti dioxide layer that favors the adhesion of osteoblastic cells, high corrosion resistance, the ability to act in redox reactions at the tissue interface, and adequate mechanical resistance [4,5]. However, primary stability is essential for successful osseointegration [6], as it favors the events necessary for bone repair, such as protein adsorption, the release of growth factors, vasoconstriction, the interaction of coagulation factors, fibrin production, and all the steps involved in bone neoformation [7].

Numerous macro- and micro-geometric modifications in dental implants in the last decades, mainly in design and surface, have been proposed and researched to promote positive effects on the osseointegration process and benefit patients who, due to some systemic alteration, may have difficulties in adequate tissue healing activity [8,9]. Systemic alterations, such as diabetes, osteoporosis, or immunosuppressive states, can impair the body’s natural regenerative processes, posing challenges to achieving successful osseointegration. By tailoring implant designs and surface characteristics to address these specific challenges, researchers and clinicians have expanded the accessibility of implant therapy to a broader population.

Regarding macro-geometric modifications, these are currently related to the goal of generating the least possible trauma on the bone tissue, that is, seeking to avoid/reduce compression zones during implant insertion [10,11]. In this sense, the inclusion of spaces and/or cavities stands out; these aim to avoid compression of the bone tissue during the insertion of implants, thus facilitating healing events [11,12,13,14]. However, maintaining the initial stability of the implant remains of fundamental importance to obtain adequate osseointegration, as the lack of bone compression is directly related to the reduction of insertion torque values.

On the other hand, the proposed changes to the surface of the implants, whether physical or chemical in nature, aim to optimize the biological interaction between the material and the biological environment, promoting better adhesion, proliferation, and cell differentiation [15,16]. These changes are designed to favor the integration of the device into the peri-implant tissue, stimulating cell growth and the formation of healthy tissue around the implant. Microscopic texturing or nanotopography on the surface can increase the contact area, facilitating cell adhesion. Likewise, chemical modifications, such as the addition of bioactive coatings (hydroxyapatite, for example), can induce specific biological responses, such as osteogenesis [15,17].

In this context, the exploration of macro- and micro-geometric modifications represents a crucial area of innovation in dental implantology. These efforts not only aim to enhance clinical outcomes but also to improve patient satisfaction and quality of life, even for individuals with compromised healing capacities. This introduction highlights the significance of ongoing research and development in this field, which continues to shape the future of dental implant technologies and their application in diverse clinical scenarios.

In view of the above, this preclinical study compared implants that present macrogeometry with healing chambers in the body with a micro-rough surface (control) and a new micro-nano rough surface (test). Thus, the main objective was to evaluate the influence of the new surface on the initial stages of osseointegration.

## 2. Materials and Methods

### 2.1. Implants, Surface and Group Formation

**Figure 1:**
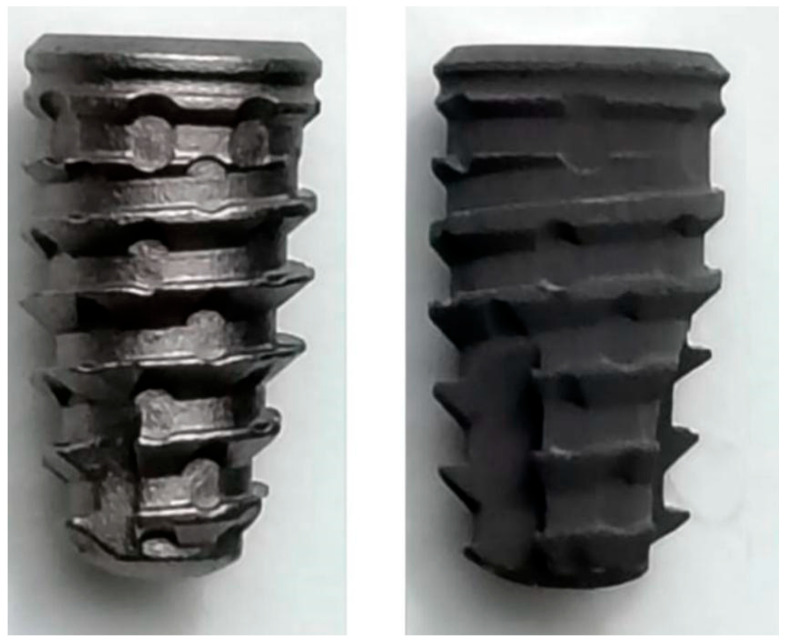
Representative image of the implants used. Left, implant of control group, and right, implant of the test group.

A total of 90 implants manufactured in pure titanium (grade IV), governed by the American Society for Testing and Material (ASTM) F67 standard [18] and produced by the company Implacil/Osstem (São Paulo, Brazil) were used. Two experimental groups were created (n = 45 implants per group): a control group, where implants with surface treatment obtained through sandblasting with titanium oxide microparticles (~150 µm) were used, and applied for approximately 20 s at a pressure of ~0.7 MPa, and a test group, where implants were sandblasted using the same process as the previous group plus acid conditioning by hydrochloric acid at a concentration of 35%, a surface called Superiore (Implacil/Osstem, São Paulo, Brazil), were used. All implants used in this study had a Morse cone connection, were conical, and had dimensions of 4 mm in diameter and 7 mm in length. Figure 1 shows a representative image of the implants used.

### 2.2. In Vitro Surface Analysis

**Figure 2:**
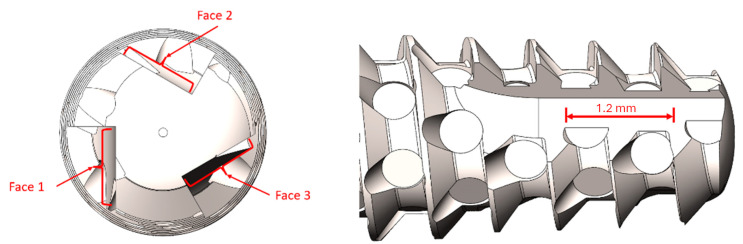
Image shows schematically where the images and roughness measurements were taken in three areas for each sample.

Five implants from each group were used to obtain images for surface analysis using a field emission gun scanning electron microscope (FEG-SEM), model Inspect 50 FEI (FEI Company, Eindhoven, The Netherlands). For the analysis of surface roughness through atomic force microscopy (AFM), model Icon PT-PKG (Bruker Optik GmBH, Ettlingen, Germany) was used. The measured parameters were the mean roughness (Ra) and the root mean square (Rq). Figure 2 shows schematically where the images and roughness measurements were taken in three areas for each sample.

### 2.3. Preclinical (In Vivo) Testing

**Figure 3:**
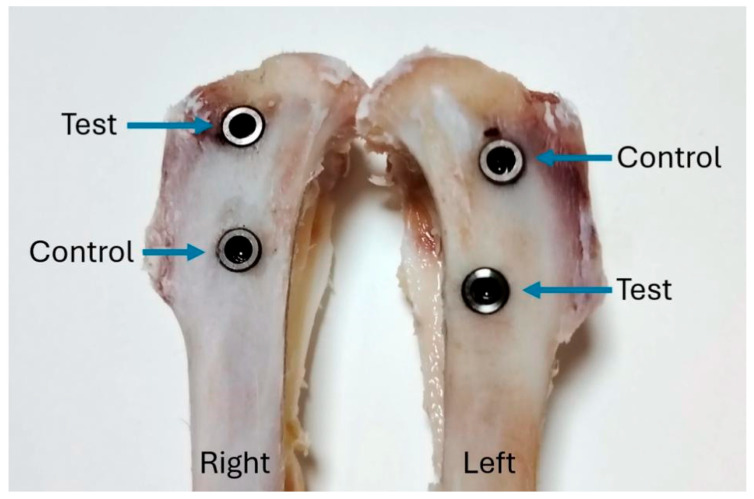
Distribution of the implant samples in the animals’ tibias.

Prior to carrying out the study, the project was evaluated by the Animal Ethics Committee of the University of Rio Verde (Brazil), being authorized under number 04/2020 on 19 April 2021. The animals received all handling, care, and treatment in accordance with the recommendations of the National Council for the Control of Animal Experimentation (CONCEA, Brasília, Brazil) [19] and were used in other studies carried out by our research group [12,13,14]. All animals were kept in individual cages, with 12 h of light, and food and water ad libitum throughout the experiment. The animals were checked twice a day by the responsible veterinarian until they had fully recovered. Medications for pain control and possible infections were administered as described in the following paragraph. A total of 80 implants were used, which were implanted in 20 adult New Zealand rabbits (Oryctolagus cuniculus), with an average weight of 4 kg. Each animal received four implants, two for each tibia. Initially, a draw was made between the right and left tibia for equal distribution of the implants between the proximal and distal portions, and then the implants were installed, as shown schematically in Figure 3.

Initially, the animals were anesthetized with a mixture in the same syringe of ketamine 35 mg/kg (Dopalen—Ceva Saúde Animal Ltd.a, Paulínia, Brazil) and xylazine 5 mg/kg (Anasedan—Ceva Saúde Animal Ltd.a, Paulínia, Brazil), administered intramuscularly (IM). Local anesthetic blockade was also performed on each tibia using Articaine 4% 1:100—(DFL, Rio de Janeiro, Brazil), applied subcutaneously (SC) to the surgical incision line. Morphine 5 mg/kg (Dimorf—Cristália Produtos Químicos Farmacêuticos Ltd.a, São Paulo, Brazil) and meloxicam 0.2 mg/kg (Maxicam—Ouro Fino Saúde Animal Ltd.a, Cravinhos, Brazil) were administered as trans-surgical analgesia, both via SC. Prior to the incision, antibiotics were also administered via SC at a dose of 84,000 IU/kg (Pentabiotic—Zoetis Industria de Produtos Veterinários Ltd.a, Campinas, Brazil). As postoperative care, all animals were housed in individual cages and monitored until full anesthetic recovery, followed by the administration of analgesics and anti-inflammatory drugs. As analgesia in the first 24 h after surgery, 5 mg/kg of morphine was administered every 6 h via SC. As anti-inflammatory medication, 0.2 mg/kg of Meloxicam via SC was administered every 24 h for 5 days. Regarding the antibiotic, as Pentabiotic has an action of up to 7 days and was applied prophylactically in the preoperative period, postoperative application was not necessary.

### 2.4. Surgical Procedures

**Figure 4:**
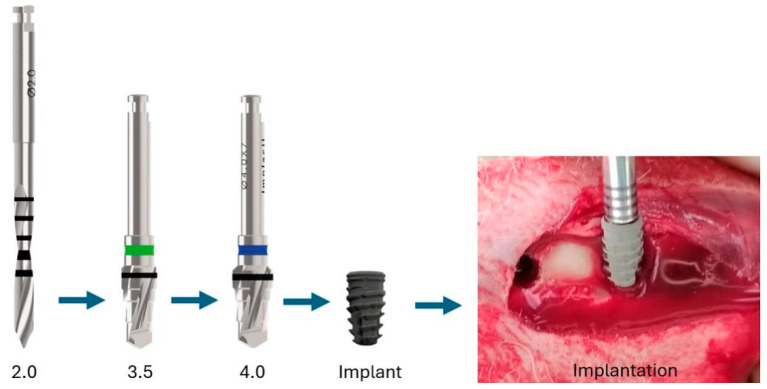
Image of the drill sequence to insert the implants for both groups into the tibia bones.

After trichotomy of the rabbits’ tibia, antisepsis of the region was performed with a povidone solution and 70% alcohol. A linear incision of ~5 cm in the proximodistal direction starting close to the joint was made on the internal portion of each tibia. The tissues were moved away, exposing the bone tissue, and the bone beds were prepared using the same sequence of burs for all implants, according to the manufacturer’s recommendations (Figure 4).

All preparations were carried out with abundant irrigation with 0.9% saline solution and a speed of 600 rpm. Subsequently, the implants were installed with different surface treatments and with the distribution previously demonstrated in Figure 3. The insertion torque of the implants was ~15 Ncm for all implants. The animals were euthanized after 3 and 5 weeks of implantations (n = 10 per time) using an overdose of anesthetics. Both tibias were collected and immediately immersed in 10% formaldehyde solution. Half of the samples from each group (n = 20), 10 for each evaluation time, were treated and evaluated histologically and histomorphometrically. In the other half of the samples, the value of the implant removal torque was evaluated for each time. Furthermore, stability values were measured by RFA for all implants and times, as described below.

### 2.5. Stability and Removal Torque Measurements

**Figure 5:**
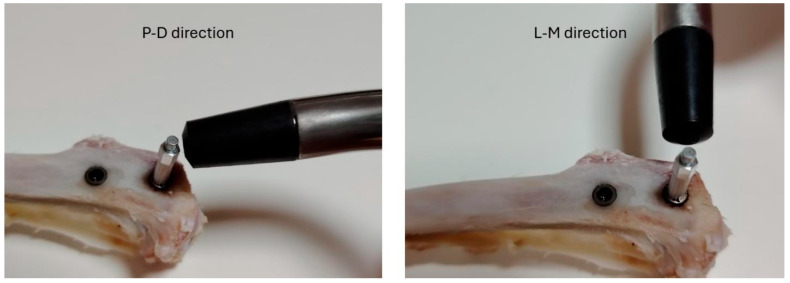
Measurements of the implant stability using the Osstell system in two directions (proximodistal (P-D) and lateromedial (L-M)).

Implant stability was measured by resonance frequency in the moment after the implant insertion (m1) and at the moment of two euthanasia times: 3 weeks (m2) and 5 weeks (m3). For this purpose, the Osstell device (Osstell AB, Gothenburg, Sweden) was used, which measures stability through magnetic resonance imaging (RFA), and these measurements are referred to as the implant stability quotient (ISQ). A SmartPeg type 26 sensor (Osstell AB, Gothenburg, Sweden) was installed in each implant, and two measurements were taken from each implant: in the proximodistal (P-D) direction and in the lateromedial (L-M) direction (Figure 5). Then, an average of the two direction measurements was calculated for each sample. It is important to emphasize that each sample was identified so that the ISQ measurements, removal torque, and histomorphometric analysis could be subsequently statistically analyzed regarding correlation and/or correspondence.

**Figure 6:**
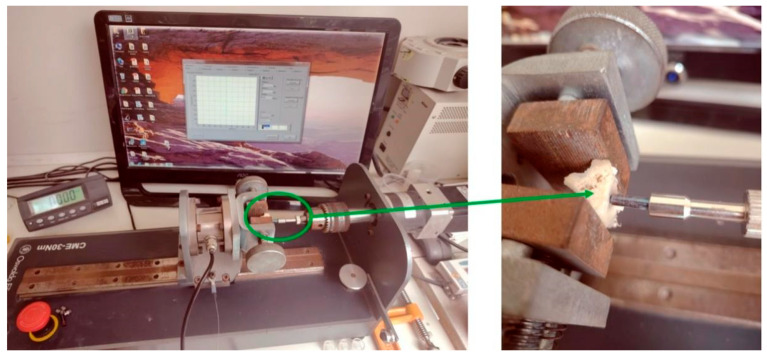
Image of the equipment used for the removal torque measurements. Green set indicates the tibia bone with the implant positioned.

Maximum torque values (peaks) were measured at implant removal at both sacrifice times (3 and 5 weeks), with 20 implants from each group being evaluated (n = 10 per time). Removal torque values were measured with a digital torque meter, model CME-30 (Osvaldo Filizola, São Paulo, Brazil), demonstrated in the Figure 6.

### 2.6. Histological Preparation and Analysis of Samples

**Figure 7:**
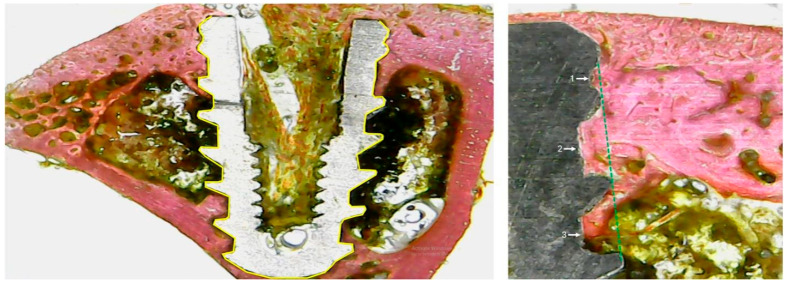
Representative images of the measurements. %BIC (**left image**), where the yellow line represents the 100% of the implant surface, and %BAFO (**right image**) in the three first threads demonstrate by the numbers 1, 2 and 3.

After seven days, all samples preserved in formaldehyde solution underwent a dehydration process using an alcohol series with increasing ethanol concentrations (50–100%). The dehydrated samples were inserted into historesin (Technovit 7200 VLC, Kultzer & Co, Wehrhein, Germany) and polymerized. The blocks containing the tibias with the implants were cut in the longitudinal direction of the implants, obtaining slices of ~70 µm, which were sanded and polished using a sequence of sandpaper from 180 to 1200 mesh. After being stained with picrosirus hematoxylin staining technique, the images were obtained using a microscope, model Nykon E200 (Tokyo, Japan). The percentage of bone-to-implant contact (%BIC) around each sample was measured, considering the surface around the entire implant as 100%. Furthermore, the bone area fraction occupancy (%BAFO) was measured in the three initial threads of each sample on each side of the implant. All of these measurements (%BIC and %BAFO) were made in ImageJ software version 1.52v (National Institute of Health, Bethesda, MD, USA). Figure 7 shows schematically how and where the measurements were made.

### 2.7. Statistical Analysis

GraphPad Prism 9 software (San Diego, CA, USA) was used for statistical analyses. The data obtained were assessed for normality using the Shapiro–Wilk test, demonstrating, in all evaluations, normal distribution (α = 0.05). Possible differences between groups at each time point were examined using the Mann–Whitney test (*p* < 0.05). The correlation between the variables ISQ vs. RTv and ISQ vs. %BIC were determined using the Pearson correlation test, with a significance level of 5%. The Bland–Altman method was used to compare the two biomechanical tests (ISQ vs. RTv), assessing the agreement between them. The mean of the differences and the limits of agreement (mean ± 1.96 standard deviations) were calculated, in addition to analyzing the dispersion of the data in a Bland–Altman plot.

The sample size was calculated based on optimal statistical power using the GPower 3.1.9.7 software (Trier, Germany). The study involved 80 implants (40 per group) across 20 rabbits (4 implants per rabbit), and animals were euthanized at two different time points (3 and 5 weeks), with 10 animals per time point. This design allowed for an adequate sample size to detect differences in implant stability, removal torque, and histomorphometric analysis. The significance level (alpha) was set at 0.05, and the target power (1 − β) was set to 0.80, meaning the study aimed for an 80% chance of detecting a true effect if one existed. Given these assumptions—effect size, variability, sample size, and power—the power calculation was designed to ensure sufficient sensitivity to detect meaningful differences, particularly in ISQ and RTv, which showed the largest differences between groups. This power value was considered adequate to ensure the accuracy of the results and the ability to detect real effects, if any, between treatments at the two time points evaluated. The differences in means, along with their respective standard deviations, were used to estimate the variability for the power calculation. The standard deviations across the groups ranged from 3.17 to 5.54, reflecting moderate variability in the outcomes.

## 3. Results

### 3.1. In Vitro Results

**Figure 8:**
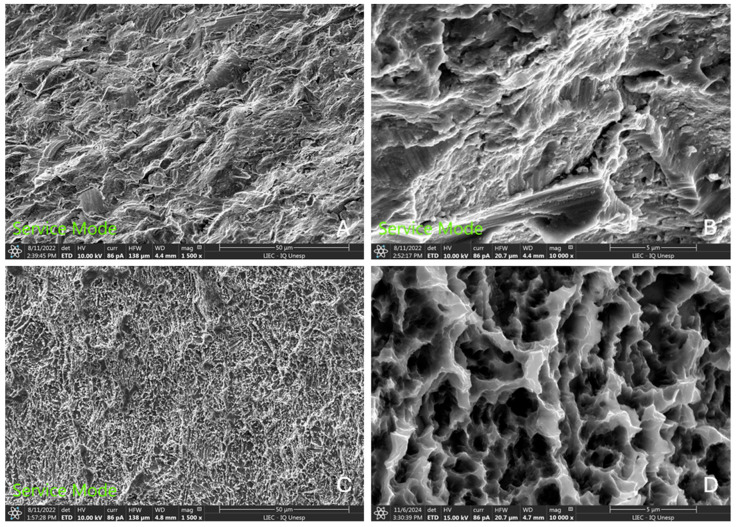
SEM images showing the surfaces of both groups. Control group (**A**,**B**) images. Test group (**C**,**D**) images. (**A**,**C**) with magnification of 1500×. (**B**,**D**) with magnification of 10,000×.

In the images obtained by SEM, it was observed that the acid conditioning after blasting with titanium oxide microparticles resulted in a surface for the test group with micro-nano porosities and a more homogeneous appearance, without the spicules and/or edges present in the control group samples. The surface of the test group presented a roughness with a peak/valley size ratio more suitable for osseointegration compared to the control group (sandblasted surface). Figure 8 shows the comparison between the surfaces of both groups at different magnifications.

**Figure 9:**
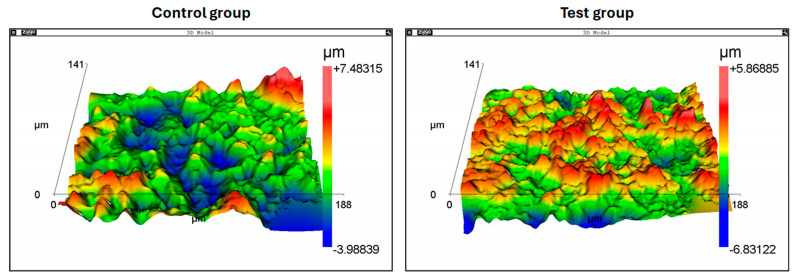
Representative images of the AFM analyses of both groups.

AFM analyses also showed a more uniform roughness pattern in the test group samples compared to the control group samples, as demonstrated in Figure 9.

Furthermore, the roughness measured for both groups is presented and statistically compared in Table 1.

### 3.2. In Vivo Results

**Figure 10:**
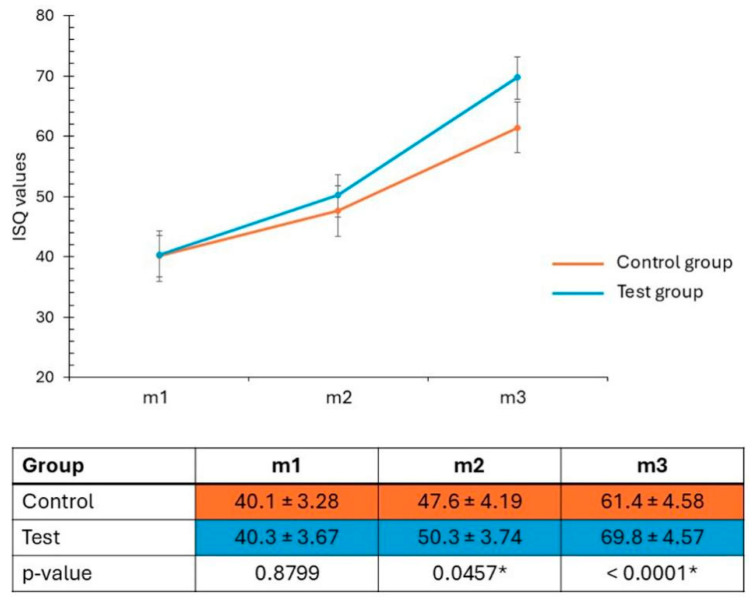
ISQ evolution of each group at each measurement time point: m1, at the implant insertion; m2, after 3 weeks; and m3, after 5 weeks of the surgeries.

All animals had an uneventful and infection-free postoperative period. All installed implants showed signs of osseointegration. The stability values measured at the three time points (initial, 3, and 5 weeks) are presented and statistically compared in Figure 10. The samples from the test group showed a faster evolution compared to the samples from the control group.

The measurements obtained in the implant removal torque test showed higher values for the test group at both times. The values obtained after 3 weeks were 44.5 ± 4.25 Ncm for the control group and 48.6 ± 3.17 Ncm for the test group, with a statistically significant difference between them (*p* = 0.0336). At 5 weeks, the values were 64.3 ± 4.50 Ncm for the control group and 76.1 ± 4.18 Ncm for the test group, with a statistically significant difference between them (*p* = 0.0002).

**Figure 11:**
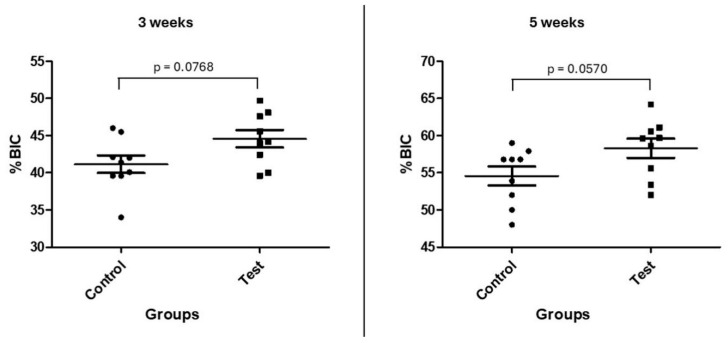
Graph showing the %BIC data distribution and statistical analysis of the groups at both times.

**Figure 12:**
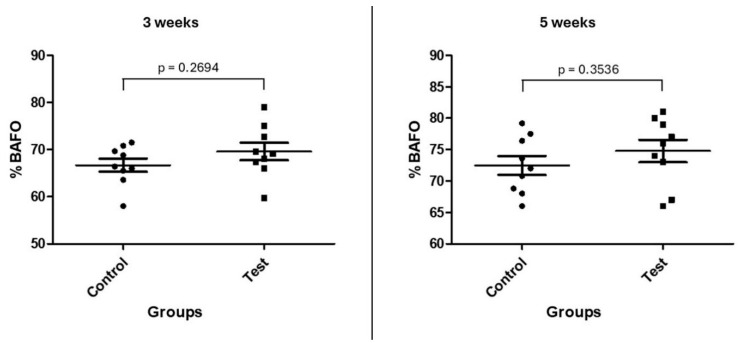
Graph showing the %BAFO data distribution and statistical analysis of the groups at both times.

The measurements obtained from %BIC and %BAFO showed similar values for both groups at both evaluation times. Figure 11 and Figure 12 show the data distribution and statistical analysis between groups.

**Figure 13:**
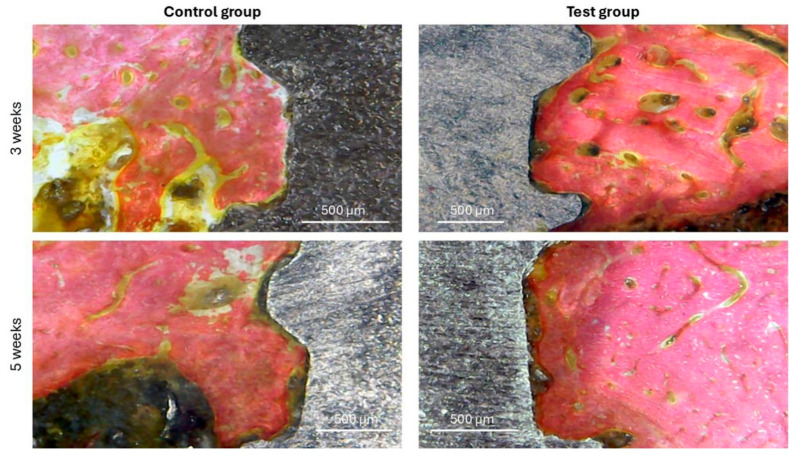
Representative histological images of both groups at both evaluation times. 100× magnification.

Furthermore, it was possible to verify, in the histological images, signs of greater stimulation and formation of bone tissue around the implants in the samples from the test group compared to the samples from the control group at both times (3 and 5 weeks), as demonstrated in Figure 13.

**Figure 14:**
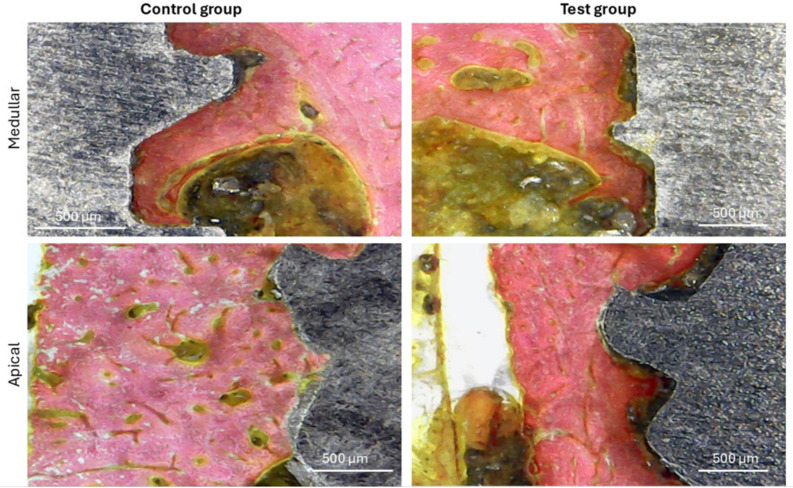
Histological images of both groups at 5 weeks show the bone formation in the medullary and apical portions, where we can observe, through the staining, a more advanced bone morphology in the test group compared to the control group. 100× magnification.

Figure 14 presents images of bone activity in both groups in different positions (medullary and apical), showing that more effective formation occurred in the test group compared to the control group, mainly after 5 weeks.

**Figure 15:**
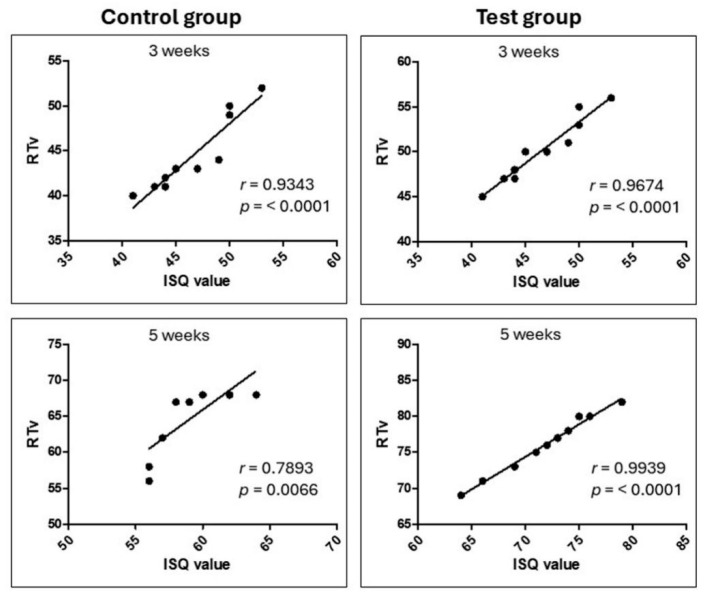
Graphs of the Pearson correlation test between ISQ values and removal torque values (RTv) in each group/time, showing a strong correlation.

The results of the Pearson correlation test revealed a very strong correlation between the two variables of ISQ and RTv. Figure 15 presents the obtained graphs and the correlation data.

**Figure 16:**
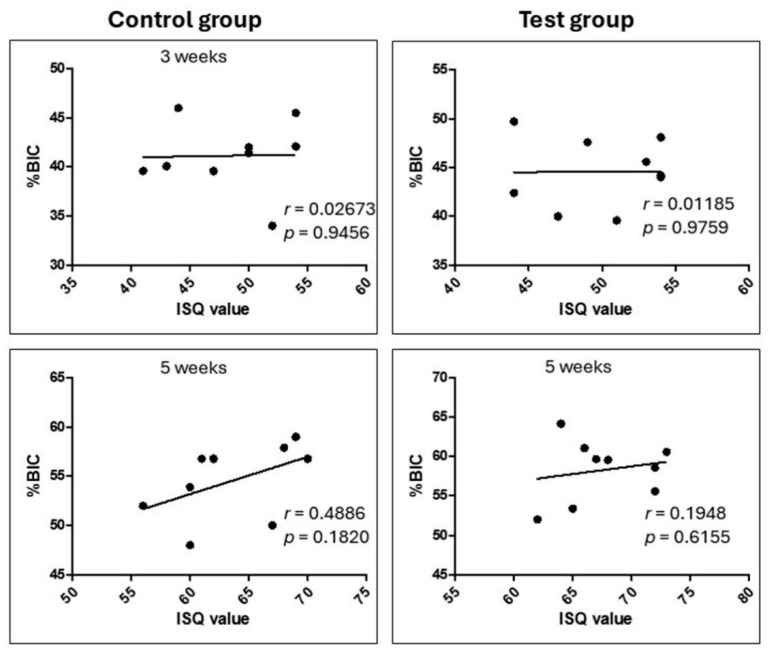
Graphs of the Pearson correlation test between ISQ values and percentage of bone to implant contact (%BIC) in each group/time, showing that there is no correlation between these parameters.

However, the analysis using the Bland–Altman method showed that, despite the high correlation, there was no significant correspondence between these variables (ISQ and RTv), with the differences observed outside the limits of agreement. On the other hand, no significant correlation was observed between the stability index (ISQ) and the percentage of bone contact with the implant (%BIC), with the data presented in Figure 16.

## 4. Discussion

This study compared two implant surfaces with the same macrogeometry (with healing chambers in the implant body), which was tested in previous studies and was shown to be more efficient for the initial osseointegration process [12,13,14]. First, in vitro tests were performed using scanning electron microscopy, atomic force microscopy, and roughness patterns. The results showed that the acid etching applied to the sandblasted surface (test group) left the surface more homogeneous and with a peak/valley size ratio more suitable for osseointegration compared to the control group (sandblasted surface). In vivo tests were performed at early stages of osseointegration (3 and 5 weeks) in rabbit tibias using RFA analysis, removal torque, and histomorphometric analysis. The results demonstrated that in the samples of the test group, the measured values of ISQ (stability) and implant removal torque were significantly higher at both evaluation times (3 and 5 weeks). However, the measurements of %BIC and %BAFO did not show differences between the groups at the two evaluation times. However, both surfaces showed significant improvements at 5 weeks compared to the analyses performed at 3 weeks. These findings suggest that the macrogeometry of the implant associated with the surface of the test group may accelerate the process in the initial phase of osseointegration.

Micro-texturing of the implant surface has been reported to improve surface topography and, consequently, may improve osseointegration levels [15,20]. In our analyses comparing both surfaces in vitro, it was possible to verify that the acid conditioning applied to the sandblasted surface (test group) left the topography more homogeneous and with more adequate roughness values compared to the surface of the control group (sandblasted), corroborating reports from other studies [20,21,22]. It is also important to highlight that the blasting applied to both groups was conducted using titanium oxide microparticles, which when compared to the more traditional blasting used by the global industry, with aluminum oxide particles, promotes better quality and less presence of contaminants on the surface [23].

The stability of implants during the osseointegration process has been described in the literature as being of fundamental importance [3,6,12,13,14]. Initial stability can be measured mechanically by insertion torque and/or through devices that use resonance frequency analysis. In our present study, insertion torque was not considered in the analyses because the same implant design was used in both groups, showing no differences in torque values. Thus, the stability analysis was performed by measuring through RFA, which showed that the implants in the test group had a faster evolution compared to the control group, especially in the samples with a time of 5 weeks. Other studies have shown similar results in the evolution of stability in implants with microtextured surfaces [21,24,25].

Although some studies have proposed other mechanical methods to evaluate the quality of newly formed bone tissue around implants, such as push-in and/or pull-out tests [26,27], in our study, we used the implant removal torque test to be able to compare the results with another study previously carried out by our group [13]. Regarding the implant removal torque, the samples from the test group presented statistically higher values for both measurement times, and after 5 weeks, the differences were significantly greater between the groups. These findings corroborate results presented in other similar studies in animals [13,28,29]. Furthermore, when we compared the data obtained in the previous study [13] with the control group that presented the same macrogeometry and surface, the results were similar, corroborating these previous findings.

Regarding the measurements of %BIC and %BAFO, the results obtained showed no differences between the two groups at either assessment time. Soares et al. (2015) [30] carried out a similar study on rabbit tibias, evaluating the same implant model with two different surfaces, and obtained similar results, that is, there was no difference between the %BIC and %BAFO values; however, differences were found in the ISQ values measured at both study times (2 and 4 weeks). These results are different from other studies where surface changes showed differences in the measured histomorphometric patterns; however, it is necessary to consider that most studies compare a treated surface with an untreated (smooth) surface or implants with different macrogeometries.

As previously described, the applied biomechanical tests, i.e., stability measurements (ISQ) and removal torque, showed differences between the groups, while the measurements of %BIC and %BAFO did not show differences. This fact leads us to conclude that there was a difference between the groups, mainly in the mineralization patterns during the early osseointegration process of the implants. According to other authors, texturing techniques in dental implants can influence the establishment of osseointegration, both for cell differentiation and the formation of the calcified bone matrix [31]. Although ISQ and RTv values showed significant improvements in the test group, %BIC and %BAFO measurements did not show significant differences between groups. This may be explained by the distinct nature of the tests: while ISQ and RTv reflect the functional stability of the implant, capturing early changes in the interaction of the implant with the bone, %BIC and %BAFO evaluate the biological process of osseointegration, which may take longer to manifest itself in a significant way. In our present study, no correlation was detected between ISQ and %BIC, corroborating the findings of other preclinical studies [32].

Furthermore, despite a strong correlation observed between ISQ and removal torque, the results of the Bland–Altman test indicate that these two methods do not present good agreement. This suggests that, although both tests are related, their measurements are not identical and may present systematic differences or variability. The strong correlation indicates that, in general, the two tests tend to behave similarly, but the Bland–Altman test reveals that discrepancies between measurements can be more significant in certain ranges of values. This may be explained by the individual limitations of each method, such as the fact that ISQ is an indirect measure of implant stability, whereas removal torque is a direct measure of the force required for removal. Despite this lack of agreement, the strong correlation between the tests still suggests that both are useful for assessing implant stability, although the choice between them depends on the clinical context and the purpose of the assessment. Future studies could investigate the causes of this discordance and seek ways to improve the accuracy and validity of the tests to ensure a more reliable assessment of implant stability.

The improved surface of the test group may offer several benefits for osseointegration, particularly in patients with systemic conditions such as osteoporosis and diabetes. In osteoporosis, where bone density is reduced and bone healing is often slower, the enhanced surface may facilitate better bone–implant contact by promoting initial bone formation and stability [33,34]. This could lead to improved osseointegration, which is crucial for implant success in these patients. Similarly, in diabetes, where impaired bone healing and vascular complications are common, the modified surface could help enhance the biological response by encouraging stronger bone-to-implant contact and promoting faster osseointegration [35]. The micro-nano-topography of the surface may stimulate cellular activity and improve bone remodeling, potentially overcoming some of the healing challenges associated with these systemic conditions [36]. Therefore, this surface modification could offer significant advantages in improving implant outcomes in patients with osteoporosis and diabetes.

Although the findings are promising, it is important to note that the study was conducted in a specific animal model (rabbit), and the results may not be fully extrapolated to humans. In addition, the follow-up time was limited to 5 weeks, and complete osseointegration may require longer follow-up to assess long-term effects. Future research could investigate the effect of different implant surfaces over a longer period of osseointegration to assess long-term stability. Furthermore, it would be interesting to conduct studies in human models or other animals with bone characteristics closer to those of humans to assess the clinical applicability of the findings. Furthermore, it would be relevant to consider discussing possible limitations of the test group, such as the long-term stability of the implant or its performance under load, even though these aspects were not directly evaluated in this study. Although surface modification showed initial benefits in terms of osseointegration and stability, assessing the durability of these effects over time is essential to understand the success of the implant in real clinical conditions.

## 5. Conclusions

In conclusion, the surface treatment presented in the test group resulted in a more homogeneous surface with a more favorable peak/valley ratio for osseointegration compared to the control group. The in vivo results obtained from early-stage osseointegration tests in rabbit tibias revealed that the test group exhibited significantly higher stability (ISQ) and implant removal torque values at both 3 and 5 weeks. While no significant differences in %BIC and %BAFO were observed between the groups, both surfaces showed considerable improvements by 5 weeks compared to 3 weeks. These findings suggest that the enhanced macrogeometry and surface properties of the test group implants could facilitate a faster osseointegration process, particularly during the early stages of healing.

## Figures and Tables

**Table 1:** Roughness data obtained in both groups and statistical analysis.

Group	Ra Values	Rq Values
Control	1.55 ± 0.16 µm	1.91 ± 0.22 µm
Test	0.95 ± 0.10 µm	1.34 ± 0.13 µm
*p*-value	0.0022 *	0.0022 *

Ra = mean roughness; Rq = the root mean square; * statistical difference (*p* < 0.05).

## Data Availability

All relevant data are contained within the paper.
